# Contemporary views on inflammatory pain mechanisms: TRPing over innate and microglial pathways

**DOI:** 10.12688/f1000research.8710.1

**Published:** 2016-09-30

**Authors:** Zhonghui Guan, Judith Hellman, Mark Schumacher

**Affiliations:** 1 Department of Anesthesia and Perioperative Care, University of California, San Francisco, CA, USA

**Keywords:** inflammation, pain, innate immunity, microglia, nociceptors

## Abstract

Tissue injury, whether by trauma, surgical intervention, metabolic dysfunction, ischemia, or infection, evokes a complex cellular response (inflammation) that is associated with painful hyperalgesic states. Although in the acute stages it is necessary for protective reflexes and wound healing, inflammation may persist well beyond the need for tissue repair or survival. Prolonged inflammation may well represent the greatest challenge mammalian organisms face, as it can lead to chronic painful conditions, organ dysfunction, morbidity, and death. The complexity of the inflammatory response reflects not only the inciting event (infection, trauma, surgery, cancer, or autoimmune) but also the involvement of heterogeneous cell types including neuronal (primary afferents, sensory ganglion, and spinal cord), non-neuronal (endothelial, keratinocytes, epithelial, and fibroblasts), and immune cells. In this commentary, we will examine 1.) the expression and regulation of two members of the transient receptor potential family in primary afferent nociceptors and their activation/regulation by products of inflammation, 2.) the role of innate immune pathways that drive inflammation, and 3.) the central nervous system’s response to injury with a focus on the activation of spinal microglia driving painful hyperalgesic states.

## Primary afferent nociceptors and inflammatory pain

Specialized primary afferent neurons that function to detect noxious chemical, thermal, and mechanical stimuli are referred to as nociceptors ^1^. Their cell bodies, found primarily in the trigeminal and dorsal root ganglion (DRG), provide sensory innervation to virtually all tissues – except the brain parenchyma. Specialized receptors, channels, and synthetic pathways help define the specificity of particular nociceptor subtypes, allowing the detection and signaling of both acute and persistent (chronic) noxious stimuli. We will focus on two principle receptors/channels that have been identified and characterized on nociceptors that detect noxious inflammatory stimuli. The first, transient receptor potential cation channel subfamily V member 1 (TRPV1 – previously known as vanilloid receptor 1 [VR1]), was initially reported to function as an integrator of multiple noxious stimuli through the demonstration that diverse products of inflammation, such as protons, anandamide, bradykinin, and nerve growth factor (NGF), functioned as positive modulators or full agonists at TRPV1 ^2, 3^. Products of the lipoxygenase pathway of arachidonic acid, 12-(S)-hydroperoxyeicosatetraenoic acid and leukotriene B4, have also been found to activate TRPV1 *in vitro*, and activated protein kinase C can directly activate or lower the activation threshold of TRPV1 to thermal stimuli ^2, 4– 8^. Two derivatives of dopamine (N-arachidonoyl dopamine and N-oleoyl dopamine) have also been found to activate TRPV1 and are associated with experimental hyperalgesia ^9, 10^ (for review, see Figure one and also 11, 12).

**Figure 1.**
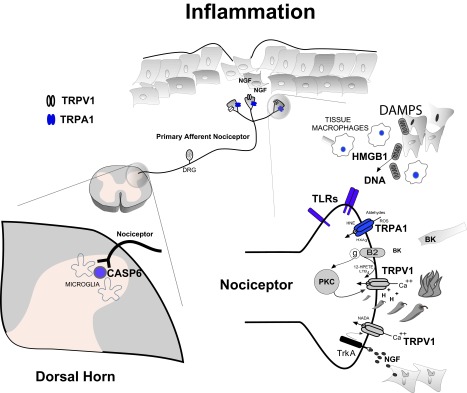
Inflammatory Pain. Tissue injury evokes a complex series of cellular responses that together is proposed to drive painful hyperalgesic states. Specialized primary afferent nociceptors (top center) innervate tissues and signal potential or actual cellular injury through detection of noxious chemical, thermal and mechanical stimuli. Electrochemical transduction of noxious stimuli at nociceptor terminals include activation of transient receptor potential (TRP) ion channel family members. As a result of the synthesis and/or release of injury – induced inflammatory products, nociceptor transducing elements may be positively modulated or directly activated driving painful and hyperalgesic states. A number of these products (eg: peptides [BK], activation of PKC, TrkA activation by NGF, acid [H ^+^], lipoxygenase products - 12-HPETE, LTB _4_, NADA, as well as reactive oxygen species [ROS], aldehydes, HNE and HXA _3_) have been shown to either modulate or activate TRPV1 and TRPA1 respectively (bottom right). Certain products of inflammation (eg: nerve growth factor [NGF], ROS, aldehydes) modulate multiple pain transducing receptors/elements. Depending on the mechanism and severity of tissue injury, innate immune cell responses will be recruited. Damage-associated molecular patterns (DAMPs) such as HMGB1 and mitochondrial derived DNA bind and activate toll-like receptors (TLRs) expressed on nociceptor terminals further driving hyperalgesia. Monocyte derived macrophages invade injured tissue and release a complex array of cytokines, chemokines and growth factors such as NGF. Together, they conspire to transform nociceptor phenotype to pathophysiologic states of persistent nociceptor activation, lowered firing thresholds and/or exaggerated response properties. Tissue inflammation also influences the central processing of nociceptive input in the dorsal horn of the spinal cord (bottom left). As a result, central nociceptor terminals upregulate and release signaling molecules such as CASP6 that activates microglia – dependent inflammatory hyperalgesia.

Taken together, it is proposed that the development of thermal hyperalgesic states, and in part spontaneous inflammatory pain, arises from the activation of TRPV1 expressed on C-type nociceptors. Moreover, the trophic factor NGF, derived from inflamed non-neuronal cells, has been found to drive both early and long-term pain behaviors ^13– 17^. In fact, long-term (days to weeks) development of thermal hyperalgesia appears to be dependent on increased expression of TRPV1 in nociceptors ^18– 22^. More recently, overexpression of TRPV1 has also been implicated in the persistent NGF-dependent inflammatory pain of oral cancer ^23^. Interestingly, links between TRPV1 and mechanical hypersensitivity pain have continued to emerge in the context of inflammation arising from pathophysiologic models of visceral/colorectal distension ^24– 26^, bone cancer pain ^27– 29^, sickle cell disease ^30^, and UVB-induced skin inflammation ^31^. Taken together, these findings also illustrate the limitations of certain models of inflammation. Notably, the experimental use of complete Freund’s adjuvant (CFA) or other agents may not necessarily induce inflammatory conditions observed in human disease.

A second transient receptor potential-related channel expressed on nociceptors, transient receptor potential cation channel subfamily A member 1 (TRPA1), was subsequently identified and has been considered by some investigators as a “gatekeeper for inflammation” ^32^. TRPA1 is now considered to play an important and possibly complementary role to TRPV1 in the development and maintenance of inflammatory pain states. This is supported by reports that TRPA1 is activated by both exogenous (allyl isothiocyanate [mustard oil], acrolein, and aldehydes) and endogenous (methylglyoxal, 4-hydroxynonenal, 12-lipoxygenase-derived hepoxilin A3, 5,6-epoxyeicosatrienoic acid, and reactive oxygen species [ROS]) inflammatory mediators ^33^. Increasingly, TRPA1 has been linked to persistent models of inflammatory pain, mechanical and cold hypersensitivity ^34^, inflammatory muscle pain ^35^, and pancreatitis pain driven by multiple inflammatory pathways ^36– 39^.

Given TRPV1 and TRPA1’s seminal roles in the signaling of inflammatory pain, there has been considerable interest in the development of high-affinity antagonists against them ^40, 41^. Indeed, there are endogenous inhibitors of TRPV1 and TRPA1, including resolvins and maresins, which are among the group of lipid mediators that are involved in resolving inflammation ^42– 44^. Preliminary reports suggest that resolvins may help to prevent or reduce inflammatory pain via transient receptor potential channels ^42, 43, 45, 46^. Although many of these compounds have been shown in preclinical studies to reduce inflammatory pain, there is concern that, owing to a broader pattern of expression of TRPV1 and TRPA1 in neuronal and non-neuronal cell types ^47^, complete inhibition of one or both channels may result in unwanted side effects such as hypothermia or inhibition of acute protective heat pain ^41^. These concerns may be heightened given reports that TRPV1 deletion enhances local inflammation and accelerates the onset of systemic inflammatory response syndrome ^48, 49^. Paradoxically, TRPV1 activation may be protective and anti-inflammatory in certain conditions, despite its peripheral activation producing neuropeptide release and neuroinflammation. Research is ongoing to devise transient receptor potential agonist/antagonist strategies that selectively block inflammatory pain without disrupting its homeostatic or acute pain protective roles. Given these challenges, perhaps a better understanding of our innate immune system’s response to injury and its subsequent role in driving inflammatory pain may provide complementary therapeutic approaches to our understanding of spontaneous and mechanical pain mediated by TRPV1 and TRPA1 ^35, 50^.

## Role of innate immune pathways

The innate immune system initiates and directs the acute inflammatory response to microbial infections and to sterile tissue injury in a multitude of disorders including sepsis, trauma, hemorrhage, cardiac arrest, vascular occlusion, organ transplantation, and injurious chemicals. Innate immune responses are triggered through the engagement of pattern recognition receptors (PRRs) by components of microorganisms known as pathogen-associated molecular patterns (PAMPs) and/or by factors released by stressed or injured host cells that are collectively known as damage-associated molecular patterns (DAMPs) ^51– 53^. The binding of PAMPs or DAMPs to their cognate PRR triggers early inflammatory responses via complex intracellular pathways involving multiple adapter proteins, interleukin-1 receptor-associated kinases (IRAKs), mitogen-activated protein kinases (MAPKs), and NFκB, which ultimately lead to the expression and/or activation of numerous inflammatory mediators, including cytokines (e.g. TNFα, IL-1β, IL-6, and IL-10), chemokines (e.g. IL-8), ROS, and adhesion molecules, and to leukocyte trafficking and activation within organs and other tissues. These responses help to acutely contain and eliminate the infection or endogenous threat, promote the development of adaptive specific immunity, and initiate the repair of injured tissues. However, in contrast to these benefits, dysregulated inflammatory responses can lead to deleterious outcomes via excessive pro-inflammatory products, the failure to resolve inflammation and restore immune homeostasis, and/or the development of immunosuppression.

PRRs have been most extensively studied in leukocytes, but they are expressed by multiple non-leukocyte cell populations including endothelial cells, cardiomyocytes, epithelial cells, and neurons ^54– 60^. Notably, PRRs expressed in cells of the nervous system, including glial cells and neurons, are postulated to contribute to a number of acute and chronic neurologic processes including, but not limited to, ischemic brain damage, Alzheimer’s disease, neuropathic pain, and other pain syndromes such as sickle cell disease ^51, 61– 73^. A number of DAMPs induce acute inflammation via PRRs and have been implicated in chronic neuropathic pain. Analogous to PRRs’ dualistic roles in systemic inflammatory conditions such as sepsis, their activation in cells of the nervous system can have beneficial effects, such as promoting neuronal repair, but, conversely, dysregulated inflammation can also have pathologic effects on the nervous system that lead to the development chronic pain.

Members of the Toll-like receptor (TLR) family and the receptor for advanced glycation end products (RAGE) are emerging as significant contributors to the pathogenesis of neuropathic pain ^72, 74– 79^. By far the most extensively studied PRRs are the TLRs, mammalian homologs of Drosophila Toll which participate in dorsoventral development and in antimicrobial defences ^80– 82^. TLRs are transmembrane proteins that are expressed at the cell surface and in endosomes and endolysosomes ^53, 81, 82^. Common microbial TLR agonists include LPS, bacterial lipoproteins, lipoteichoic acid, peptidoglycan, flagellin, and nucleic acids ^81, 83– 90^. Endogenous agonists of the TLRs include HMGB1 (TLR2, TLR4, and TLR9), heparan sulfate (TLR4), heat shock proteins (TLR2 and TLR4), hyaluronan (TLR2 and TLR4), versican (TLR2), RNA (TLR3), mitochondrial DNA (TLR9), and β-amyloid (TLR2 and TLR4) ^61, 91– 101^. TLRs and downstream signaling intermediaries, such as the adapter proteins MyD88 and TRIF, have also been reported to contribute to neuropathic pain syndromes ^74– 76, 102, 103^. RAGE is a multi-ligand member of the immunoglobulin superfamily that is expressed at the cell surface and in a secreted form ^104^. There are numerous endogenous RAGE agonists, including, but not limited to, β-amyloid, HMGB1, and S100 proteins, and there is accumulating evidence that RAGE is important in neuropathic pain ^99, 101, 104– 109^. Notably, HMGB1 has been reported by a number of groups to be released by stressed and injured tissues and to facilitate the development of neuropathic pain ^63, 77, 78, 110– 112^. In addition to the TLRs and RAGE, other PRRs may also contribute to inflammatory pain. For example, the NLRP3 inflammasome, a multiprotein cytosolic complex responsible for the production of active IL-1β and IL-18, has been implicated in chronic pain and has been reported to contribute to opioid-induced hyperalgesia in animal models ^113– 116^. Multiple factors stimulate the NLRP3 inflammasome, including microbial components such as LPS, nigericin, zymosan, and malarial hemozoin, and several endogenous factors, including β-amyloid, uric acid, ATP, and calcium pyrophosphate dehydrate ^52, 117– 121^.

Over the last decade and a half, strong links have been identified between the nervous system and the immune system. Multiple cell lineages in the central and peripheral nervous system express PRRs, including neurons, microglia, astrocytes, Schwann cells, and oligodendrocytes ^72, 73, 122– 125^. The links between the immune system and nervous system are bidirectional – the immune system is able to modulate neuronal function and vice versa. There is strong evidence that a neuroimmune response that is mediated through the vagus nerve, spleen, and cholinergic receptors modulates host responses to endotoxemia and infection ^126, 127^. Furthermore, several studies suggest that TRPV1 modulates the outcomes of bacterial sepsis ^128– 131^. There is also accumulating evidence that the activation of innate immune pathways, particularly TLR- and RAGE-dependent pathways, contributes to the development of chronic pain following nerve injury ^62– 64, 67, 69, 79, 109, 132^. From a mechanistic standpoint, leukocyte-derived factors released in response to DAMP-mediated activation of PRRs expressed by microglia and peripheral monocytes are believed to induce pain through their actions on sensory neurons.

Intriguingly, the direct activation of neuronally expressed PRRs may also be involved in the development of acute and chronic pain. TLR agonists have been reported to directly activate DRG neurons and to increase levels of TRPV1 expression in DRG neurons ^73^. Furthermore, TRPV1-expressing nociceptive neurons have also been reported to express TLR4 ^125^. While the focus of this discussion has been on innate immune pathways in the pathogenesis of pain, recent reports also point to a role for the adaptive immune system in chronic pain ^102, 133– 137^. For example, modulating T lymphocyte cell responses pharmacologically has been reported to reduce chronic neuropathic allodynia and chronic constriction injury-induced neuropathic pain in rats ^133, 134^. Similarly, the downregulation of IL-12p70 (a proinflammatory cytokine that promotes the proliferation of T lymphocytes and natural killer cells), the deletion of the adapter protein MyD88, or the downregulation or neutralization of IL-17A (which links innate and adaptive immunity) have all been reported to attenuate chronic neuropathic pain in rodents ^102, 134, 137, 138^. The fact that diverse conditions, including chronic pain, sepsis, trauma, and ischemia reperfusion injury, have shared pathways raises the intriguing but complex possibility of developing therapeutics that can reverse inflammatory pain without compromising immune function.

## The central nervous system’s response to injury

The spinal cord microglia, the tissue-resident immune-like macrophages of the central nervous system ^139^, can respond to peripheral injuries that are distant from the spinal cord to produce neuroinflammation in the central nervous system ^140^. Indeed, traumatic injuries to the peripheral nerves activate microglia, both in the dorsal horn where sensory nerve endings from the DRG terminate and in the ventral horn where activated microglia wrap around the injured motoneurons ^141^. In fact, neuroinflammation in the spinal cord, presented as microglia activation, is well known to contribute to the development of neuropathic pain after nerve injury ^140– 143^.

One of the first clues that microglia might contribute to inflammatory pain came from the report that spinal cord microglia are activated in the formalin inflammatory pain model ^144^. In this widely used inflammatory pain model, 5% formalin is injected subcutaneously into the hind paw of a rat or mouse. Fu *et al*. observed spinal cord microglia activation, defined as enhanced immunoreactive signaling of microglia markers, after formalin injection in male rats, starting on day 1 and peaking on day 7 post injection ^143^. Interestingly, pre-treatment of local anesthetic bupivacaine does not block formalin-induced spinal cord microglia activation, even though it successfully blocks formalin-evoked pain behaviors ^145^, indicating that the nociceptive input from the acute inflammatory response of formalin is not required for spinal cord microglia activation.

Subsequently, it was reported that p38 MAPK is activated in the spinal cord microglia after formalin injection in male rats ^146^, and this activation of p38 MAPK occurs in 2 phases ^147^. The first phase of microglial p38 activation starts quickly, just a few minutes after formalin injection, and lasts for 1 hour, the time course that correlates with early acute spontaneous nociceptive behavior ^146, 147^. Indeed, intrathecal inhibition of microglia with minocycline greatly attenuates formalin-evoked acute flinching behavior ^148^. The second phase of microglial p38 activation starts 1 day after formalin injection and lasts for 7 days, the time course that correlates with persistent mechanical hypersensitivity induced by formalin injection ^147^. Inhibition of p38 kinase attenuates both acute nociceptive behavior and persistent mechanical hypersensitivity induced by formalin injection ^146, 147^. In fact, there are two p38 isoforms in the spinal cord, with p38α expressed in neurons and p38β expressed in microglia ^149^. Downregulation of microglial p38β, rather than neuronal p38α, attenuates formalin injection-induced acute nociceptive behavior ^149^. In addition to p38 MAPK, Src family kinase (SFK) is also activated in spinal cord microglia, starting 1 day after formalin injection and lasting for 7 days ^150^. Unlike p38 MAPK, SFK is necessary for persistent mechanical hypersensitivity after formalin injection, although it is not required for formalin-induced acute spontaneous nociceptive behavior ^150^.

Recent evidence further supports the idea that formalin injection produces early microglial activation ^151^. Berta *et al*. demonstrated that within 30 minutes of formalin injection, caspase-6 (CASP6) is upregulated in the central terminals of primary afferents and is released in the spinal cord ^151^. The resultant CASP6-mediated cascade activates spinal cord microglia and stimulates microglial TNF-α synthesis and release through p38 and ERK kinases. In fact, formalin-induced second-phase inflammatory pain is CASP6 dependent, and intrathecal injection of CASP6 or CASP6-treated microglia produces pain behavior mediated in part through stimulation of spinal cord lamina II neurons. Moreover, CASP6 is also required for capsaicin-elicited secondary mechanical hypersensitivity as well as bradykinin, carrageenan, and CFA-induced inflammatory pain. As TRPA1 is one of the receptors targeted by formalin ^152^, it is likely that in the formalin inflammatory pain model, formalin activates DRG neurons through TRPA1 to induce CASP6 and subsequently activates spinal cord microglia shortly after formalin injection.

Although spinal cord microglia are clearly activated shortly after the formalin injection in the hind paw, whether the long-term microglia activation days after formalin injection is caused by tissue inflammation itself is controversial. Importantly, in addition to tissue inflammation, hind paw formalin injection also produces damage to peripheral nerve endings, as transcription factor ATF3, a marker for peripheral nerve injury ^153^, is induced in DRG neurons after formalin hind paw injection ^154^. Given that peripheral nerve injury is a well-known factor that activates spinal cord microglia to produce pain behaviors ^140– 143^, it is likely that peripheral nerve injury and tissue inflammation, together, are responsible for the spinal cord microglia activation after formalin hind paw injection.

## Summary

Inflammatory pain constitutes an ongoing enigma for the development of novel analgesic agents. Despite the robust characterization of peripheral nociceptive channels (e.g. TRPV1 and TRPA1) capable of detecting a wide range of inflammatory stimuli, clinically relevant antagonists may surreptitiously disrupt essential homeostatic and protective functions such as TRPV1-dependent core temperature regulation or the detection of warmth. Time will tell if antagonists to TRPA1 will encounter similar sensory physiologic limitations surrounding their role in cold detection, mechanosensation, or cellular signaling. If systemic administration of transient receptor potential antagonists continues to be problematic, perhaps restricting these agents to peripheral and/or spinal targets could still provide the desired effect. Detailed examination of innate immune response elements holds additional promise for novel analgesic development in the treatment of inflammatory pain. For example, the role of the endogenous TLR4 and RAGE agonist HMGB1, a molecule previously associated with sepsis, now has emerged as an important participant in mediating inflammatory and neuroinflammatory pain states. Developing strategies around the blockade of HMGB1 and/or dampening overexpression of TLR4 or RAGE are plausible directions. Central spinal processing of nociceptive signaling can be modulated by microglia, the immune-like macrophage of the central nervous system, and recent evidence suggests that activated microglia also contribute to the pain produced by tissue inflammation. Further studies on the blockade of spinal CASP6 under painful pathophysiologic conditions such as bone cancer pain, sickle cell disease, or inflammatory bowel disease may represent another important therapeutic opportunity in analgesic development.

## Abbreviations

CASP6, caspase 6; CFA, complete Freund’s adjuvant; DAMP, damage-associated molecular pattern; DRG, dorsal root ganglion; IRAK, interleukin-1 receptor-associated kinase, MAPK, mitogen-activated protein kinase; NGF, nerve growth factor; PAMP, pathogen-associated molecular patterns; PRR, pattern recognition receptor; RAGE, receptor for advanced glycation endproducts; ROS, reactive oxygen species; SFK, Src family kinase; TLR, Toll-like receptor; TRPA1, transient receptor potential cation channel subfamily A member 1; TRPV1, transient receptor potential cation channel subfamily V member 1.
